# MultiContrast Delayed Enhancement (MCODE) improves detection of subendocardial myocardial infarction by late gadolinium enhancement cardiovascular magnetic resonance: a clinical validation study

**DOI:** 10.1186/1532-429X-14-83

**Published:** 2012-11-30

**Authors:** W Patricia Bandettini, Peter Kellman, Christine Mancini, Oscar Julian Booker, Sujethra Vasu, Steve W Leung, Joel R Wilson, Sujata M Shanbhag, Marcus Y Chen, Andrew E Arai

**Affiliations:** 1 Advanced Cardiovascular Imaging Laboratory, Cardiovascular and Pulmonary Branch, National Heart, Lung and Blood Institute (NHLBI), National Institutes of Health (NIH), Department of Health and Human Services, Bethesda, MD, USA; 2 Johns Hopkins Suburban Hospital, Bethesda, MD, USA

**Keywords:** Late gadolinium enhancement, Myocardial infarction, MultiContrast Delayed Enhancement, Cardiovascular magnetic resonance

## Abstract

**Background:**

Myocardial infarction (MI) documented by late gadolinium enhancement (LGE) has clinical and prognostic importance, but its detection is sometimes compromised by poor contrast between blood and MI. MultiContrast Delayed Enhancement (MCODE) is a technique that helps discriminate subendocardial MI from blood pool by simultaneously providing a T2-weighted image with a PSIR (phase sensitive inversion recovery) LGE image. In this clinical validation study, our goal was to prospectively compare standard LGE imaging to MCODE in the detection of MI.

**Methods:**

Imaging was performed on a 1.5 T scanner on patients referred for CMR including a LGE study. Prospective comparisons between MCODE and standard PSIR LGE imaging were done by targeted, repeat imaging of slice locations. Clinical data were used to determine MI status. Images at each of multiple time points were read on separate days and categorized as to whether or not MI was present and whether an infarction was transmural or subendocardial. The extent of infarction was scored on a sector-by-sector basis.

**Results:**

Seventy-three patients were imaged with the specified protocol. The majority were referred for vasodilator perfusion exams and viability assessment (37 ischemia assessment, 12 acute MI, 10 chronic MI, 12 other diagnoses). Forty-six patients had a final diagnosis of MI (30 subendocardial and 16 transmural). MCODE had similar specificity compared to LGE at all time points but demonstrated better sensitivity compared to LGE performed early and immediately before and after the MCODE (p = 0.008 and 0.02 respectively). Conventional LGE only missed cases of subendocardial MI. Both LGE and MCODE identified all transmural MI. Based on clinical determination of MI, MCODE had three false positive MI’s; LGE had two false positive MI’s including two of the three MCODE false positives. On a per sector basis, MCODE identified more infarcted sectors compared to LGE performed immediately prior to MCODE (p < 0.001).

**Conclusion:**

While both PSIR LGE and MCODE were good in identifying MI, MCODE demonstrated more subendocardial MI’s than LGE and identified a larger number of infarcted sectors. The simultaneous acquisition of T1 and T2-weighted images improved differentiation of blood pool from enhanced subendocardial MI.

## Background

The introduction of late gadolinium enhancement (LGE) as a means to identify myocardial infarction (MI) and predict recovery of left ventricular systolic function was a key factor in pushing cardiovascular magnetic resonance (CMR) into mainstream clinical use [1]. The ability to accurately detect MI, as well as characterize the transmural and circumferential extent of infarction, plays an important role in assessing viability.

MI as documented by LGE has prognostic significance [2,3]. Atypical patterns of LGE within cardiomyopathic processes such as hypertrophic cardiomyopathy and nonischemic dilated cardiomyopathy are also thought to be associated with arrhythmias [4,5], as well as increased mortality [6].

To improve clinical workflow efficiency, many imaging sites perform LGE imaging [1,7] approximately 10 min after administering gadolinium contrast. However, the contrast between the blood pool and infarction may not be optimal so early after contrast administration [8]. There is a concern that lack of contrast at the tissue-blood interface on LGE images could increase the rate of false negative LGE images in the detection of subendocardial MI.

MultiContrast Delayed Enhancement (MCODE) [9] is a CMR technique designed to help discriminate subendocardial MI from blood pool. The MCODE sequence generates both a T2-weighted image and a T1-weighted LGE image during the same breath-hold and at the same phase of the cardiac cycle. The resultant image pair can be displayed side-by-side or superimposed. Myocardium, whether normal or infarcted, can be differentiated from fluid using T2 contrast, which is minimally affected by the presence of gadolinium contrast agents when a sequence with negligible T1-weighting is employed. The T2-weighted image depicts infarcted and viable myocardial tissue with similar signal intensity, and both have better contrast with the blood pool.

In this clinical validation study, our goal was to prospectively assess whether the T2- weighted image acquired during an MCODE acquisition adds diagnostic value to the LGE images. We hypothesized that MCODE should significantly decrease false negative LGE images for diagnosing MI in both acute and chronic MI when contrast between blood and infarct is poor and the transmural extent of infarction is subendocardial.

## Methods

The study was approved by the Institutional Review Board, and all subjects provided informed consent. Imaging was performed on either a 1.5 Tesla Siemens Espree or Avanto MRI scanner (Siemens Medical, Erlangen, Germany). Seventy-three patients with a variety of diagnoses referred for CMR that included LGE were imaged (37 stress, 22 viability, 12 other). Patients with hypertrophic cardiomyopathy were excluded from analysis (n=2). Clinical data were obtained through patient history and medical records. Patients were categorized as having an MI if they had: 1) a history of an acute chest pain syndrome with associated abnormal cardiac enzyme elevation 2) evidence of MI by Q waves on EKG with angiographically significant coronary artery disease or an abnormal nuclear perfusion study, or 3) fixed perfusion defect on a nuclear study. Other patients were categorized as not having an MI.

### Image acquisition

All patients underwent cine imaging of the heart, using steady-state free precession techniques in a volumetric short-axis stack with standard three-, two-, and four-chamber long axis views. Typical parameters for the cine imaging included a matrix size of 256 × 144, slice thickness of 6mm, TE 1.65, bandwidth of 977 Hz/Px, and a flip angle of 50°. After administration of 0.15-0.2 mmol/kg gadolinium-diethylenetriamine pentaacetic acid (Gd-DTPA) (Magnevist, Berlex, Wayne, New Jersey, United States), a stack of short-axis and three standard long axis LGE images were acquired using a phase sensitive inversion recovery (PSIR) [10] spoiled gradient recalled echo sequence. The typical parameters were a matrix size of 256 × 144, 6mm slice thickness, TI individualized to null the myocardium, TE 3.25msec, TR 8.2 ms, bandwidth of 140 Hz/pixel, and an excitation flip angle of 25°. PSIR LGE was a breath-held, ECG triggered, segmented acquisition with inversions every 2 R-R intervals, acquiring a proton density (PD) weighted image on alternate heartbeats. Typical segmentation was 21 phase encode lines per heartbeat at a nominal 60 beats per minute heart rate, corresponding to a breath-hold duration of 10 heartbeats including 2 discarded beats.

**Figure 1:**
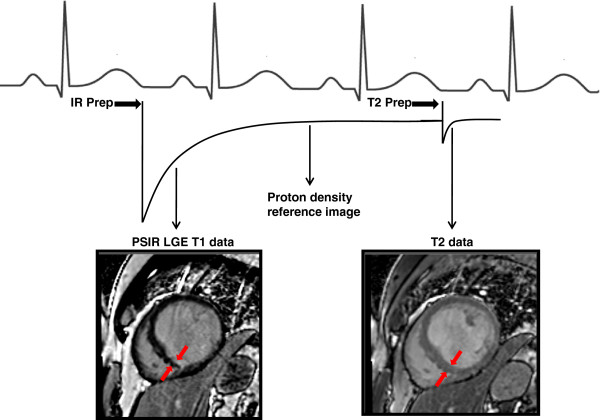
**Simplified schematic of what data is acquired in MCODE: Within the same acquisition, MCODE produces both a LGE T1 image and a T2-weighted image at similar time points in the cardiac cycle.** The LGE image is comparable to conventional methods with nulled, normal myocardium and bright MI. The T2-weighted image easily differentiates fluid (blood) from solid tissue (myocardium) but has minimal T1-weighting. Thus, the MI looks comparable to viable myocardium, and the endocardium is better delineated than on LGE images. Red arrows indicate the location of a MI on both the LGE T1 image (left) and the T2-weighted image (right).

Prospective comparisons between MCODE and standard PSIR LGE imaging were done by targeted, repeat imaging of select slice locations. A standard PSIR LGE image was obtained, followed by an MCODE acquisition and another standard PSIR LGE image on three sequential breath holds. The rationale behind repeating the standard PSIR LGE imaging was to minimize differences in image contrast attributable to renal clearance and therefore primarily compare the MCODE LGE T1 and T2 images with conventional PSIR LGE. The MCODE acquisition resulted in PSIR LGE and T2-weighted images within the same breath-held acquisition. Typical imaging parameters of the MCODE sequence included a matrix of 256 × 119, corresponding to a spatial resolution of 1.3 × 2.3 mm^2^ for a nominal 360 × 270 mm^2^ field of view, slice thickness 6 mm, TI optimized on an individual basis (but commonly 300 ms), TE 2.47 ms, TR 6.4 ms, BW 201 Hz/pixel, spoiled GRE read-out, excitation flip angle 25° for the T1-weighted PSIR LGE image and 15° for the T2-weighted image. MCODE was a breath-held, ECG triggered, segmented acquisition with inversions every 3 R-R intervals, acquiring an IR, PD, and T2-weighted image on alternate heartbeats. Typical segmentation was 30 phase encode lines per heartbeat at a nominal 60 beats per minute heart rate, corresponding to a breath-hold duration of 15 heartbeats including 3 discarded beats. The effective TE for the T2-weighted images was 40 ms. Using the segmented FLASH read-out leads to a minor T1-weighted contrast which has been found to be negligible for this application of discriminating blood pool from myocardium [9]. Figure 1 illustrates a simplified schematic of the data acquired with MCODE. The red arrows indicate an inferior wall MI that has poor infarct to blood pool contrast on the LGE T1 image on the left. In the same region, on the right panel, the red arrows point to where the subendocardial blood pool interface is clearly seen.

### Image analysis

Each image, within the standardized image set - the initial late gadolinium enhancement images (LGE1), the repeated late gadolinium enhancement images (LGE2), the MCODE (containing a PSIR LGE image and a T2-weighted image), and the third late gadolinium enhancement images (LGE3) - were read on different days from other images of the same patient by a single Level III cardiovascular magnetic resonance imaging cardiologist with over 10 years’ experience. All PSIR LGE images were categorized as to whether or not MI was present and whether a MI was transmural (>50 of the transmural extent of the myocardium) or subendocardial (≤50% the transmural extent of the myocardium). The MCODE images were reviewed independently from the standardly acquired LGE images. During the MCODE analysis, the LGE and T2 images were reviewed both side by side, as well as in a superimposed “flicker”-mode.

For images demonstrating MI, additional analysis was performed. The number of sectors with MI on LGE2 and the MCODE LGE/T2 image pair were individually tallied to determine the extent of the infarction. In all patients with MI, on the MCODE LGE T1 image, regions of interest were drawn within normal myocardium, around myocardial infarction, and within the blood pool. From the T1 image, the same regions of interest for the normal myocardium and MI were copied and applied to the MCODE T2 image. Signal intensities were reported as mean ± standard deviation.

### Statistical analysis

Statistical significance was analyzed using MedCalc Version 12.0.1 statistical software (MedCalc Software, Mariakerke, Belgium). Descriptive data are reported as mean ± standard deviation (SD) if data were normally distributed or median with interquartile range if not normally distributed. A D’Agostino Pearson test was used to determine if continuous data were normally distributed. A *t*-test was used to compare mean values of normally distributed data. A Wilcoxon test was used to compare paired categorical data or data that were not normally distributed. A McNemar test was used to compare pairs of correlated proportions. A Friedman test was used to detect differences in repeated measures for data that were not normally distributed. A Mann–Whitney test was performed on unpaired categorical data or continuous data that were not normally distributed. A weighted statistic score and corresponding p values described by Kosinski [11] were used to compare positive and negative predictive values. Statistical significance was defined as a p value < 0.05.

## Results

### Patient characteristics

The final data set consisted of 71 patients who were imaged using the multi-technique MCODE/LGE protocol. The enrolled patients represented a mixture of patients with known coronary artery disease (CAD) (54%) or intermediate to high likelihood of coronary disease with 30% (21 patients) having greater than three TIMI risk factors. Fifty-two of the 71 patients were male, and the mean age was 57.9 ± 11.1 years. Thirty-one percent of the patients were specifically referred for assessment of viability (including 12 acute and 10 chronic MI’s), while another 52% were referred for assessment of ischemia. The remaining patients were referred for indications such as assessment for nonischemic cardiomyopathy (7 patients, 10%), aortic assessment (3 patients, 4%), and congenital assessment (2 patients, 3%).

Within the group, 46 patients were categorized with MI (30 subendocardial and 16 transmural) and 25 patients without infarction. The 46 MI’s included 35 patients who had outside confirmation of a clinical event with a chest pain syndrome and cardiac enzyme abnormality and nine patients who had evidence of Q waves on EKG with angiographic evidence of CAD or an abnormal nuclear stress test. Two patients did not have a prior history of MI but had fixed nuclear defects consistent with MI.

Among the non-MI group, no one had a clinical history of an acute coronary syndrome nor evidence of Q waves by EKG.

Compared to the patients without MI, the MI subgroup of patients were more likely to have a prior history of CAD, hypertension, hyperlipidemia, and diabetes and had lower ejection fractions. Baseline characteristics are summarized in Table 1.

**Table 1:** Baseline Patient Characteristics

**Characteristic**	**All patients n=71**	**MI n=46**	**No MI n=25**	**P value**
Age - years				
Mean ± standard deviation	57.9 ± 11.1	59.9 ± 9.9	54.2 ± 12.6	0.04
Maximum, minimum	28, 83	38, 83	28,80	
Male sex – no (%)	52 (73)	35 (76)	17 (68)	0.47
CAD Risk Factors – no (%)				
Family history	12 (17)	8 (17)	4 (16)	0.89
Hypertension	49 (69)	37 (80)	12 (48)	0.006
Dyslipidemia	49 (69)	40 (87)	9 (36)	< 0.0001
Diabetes	15 (21)	14 (30)	1 (4)	0.012
Smoking	27 (38)	20 (57)	7 (28)	0.21
Known CAD – no (%)	38 (54)	37 (80)	1 (4)	<0.0001
>3 CAD Risk Factors- no (%)	21 (30)	18 (39)	3 (12)	0.002
Medications (%)				
Anti-platelets/anti coagulants				
Aspirin	49 (69)	41 (89)	8 (32)	<0.0001
Clopidogrel/Prasugrel	18 (25)	18 (39)	0 (0)	0.0004
Warfarin	4 (6)	3 (7)	1 (4)	0.69
Anti-hypertensives				
Beta blocker	48 (68)	39 (85)	9 (36)	<0.0001
Calcium channel blocker	8 (11)	5 (11)	3 (12)	0.89
ACE inhibitor	28 (39)	21 (46)	7 (28)	0.16
ARB	9 (13)	8 (17)	1 (4)	0.12
Diuretic	15 (21)	9 (20)	6 (24)	0.67
Long acting nitrates	6 (8)	6 (13)	0 (0)	0.08
Lipid Medications				
Statin	44 (62)	38 (83)	6 (24)	< 0.0001
Other lipid therapy	8 (11)	5 (11)	3 (12)	0.89
Diabetes Medications				
Insulin	7 (10)	7 (15)	0 (0)	0.052
Oral diabetes agents	11 (16)	10 (22)	1 (4)	0.059
No Cardiovascular Medications	9 (13)	1 (2)	8 (32)	0.0002
Left ventricular ejection fraction %				
Median, interquartile range	56, 15	51, 20	60,8	0.002
Indication for scan (%)				
Ischemia Assessment with Stress	37 (52)	22 (48)	15 (60)	0.34
Viability	22 (31)	22 (48)	0 (0)	<0.0001
Other	12 (17)	2 (4)	10 (40)	0.0002
Nonischemic cardiomyopathy	7 (10)	1 (2)	6 (24)	
Aorta	3 (4)	1 (2)	2 (8)	
Congenital	2 (3)	0 (0)	2 (8)	

**Figure 2:**
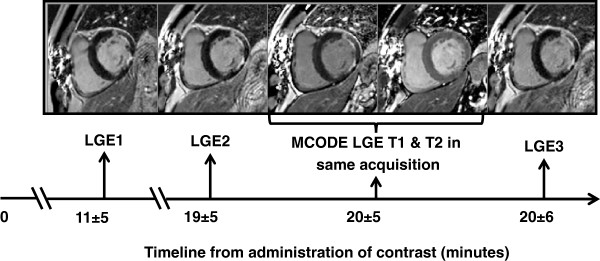
Results: Timeline of image acquisition post-contrast: The mean time elapsed after contrast administration for each acquisition is summarized.

The mean time between contrast administration and the LGE1 image was 11±5 min. The mean time between contrast administration and the LGE2 image was 19±5 min. The mean time between the contrast and the MCODE image was 20±5 min. On average, the MCODE acquisition was performed 50 s after the LGE2 and 46 s before the LGE3. The mean time between contrast administration and the LGE3 image was 20±6 min. Figure 2 summarizes the timeline of the imaging protocol.

### CMR findings

#### Diagnostic performance

Tables 2a-d and 3 illustrate the diagnostic performance of each of the imaging techniques in identifying myocardial infarction on a per patient basis. Both standard LGE and MCODE performed well in the identification of MI. MCODE had 100% sensitivity for the detection of MI with no false negatives and 88% specificity against the clinical definition of MI. LGE1 detected 38 of 46 patients with MI (sensitivity 83%), but otherwise had the same results as LGE2 and LGE3. LGE2 and LGE3 had identical results. LGE2 had a sensitivity of 85% because it missed seven MI’s. The specificity of LGE2 was 92%. Interestingly, the two LGE false positive MI’s were two of the three patients labeled as false positive MI by MCODE. MCODE had better sensitivity than LGE1 (p = 0.008), LGE2 (p = 0.02), and LGE 3 (p = 0.02) but was not statistically different in specificity.

**Table 2:** a-d: Diagnostic performance of each imaging component in detecting MI: 2 × 2 contingency tables

**a**	**MI (+)**	**MI (-)**
**MCODE (+)**	46	3
**MCODE (-)**	0	22
**b**	**MI (+)**	**MI (-)**
**LGE1 (+)**	38	2
**LGE1 (-)**	8	23
**c**	**MI (+)**	**MI (-)**
**LGE2 (+)**	39	2
**LGE2 (-)**	7	23
**d**	**MI (+)**	**MI (-)**
**LGE3 (+)**	39	2
**LGE3 (-)**	7	23

**Table 3:** Diagnostic performance of each imaging component in detecting MI: Sensitivity, Specificity, Positive Predictive Value, Negative Predictive Value

	**LGE1**	**LGE2**	**MCODE**	**LGE3**
Sensitivity*	83%	85%	100%	85%
Specificity**	92%	92%	88%	92%
Positive predictive value (PPV)**	95%	95%	94%	95%
Negative predictive value (NPV)*	75%	77%	100%	77%
Accuracy	86%	87%	96%	87%

*Sensitivity of LGE1 compared to MCODE, p = 0.008; NPV comparison, p = 0.005.

*Sensitivity of LGE2/LGE3 compared to MCODE, p = 0.02; NPV comparison, p = 0.009.

**Specificity and PPV of LGE1/LGE2/LGE3 compared to MCODE, p = not statistically significant.

LGE1, LGE2, LGE3, and MCODE identified all transmural MI’s. All MI’s missed by LGE were in subendocardial MI’s. When analyzing subendocardial MI, LGE1 images identified 22 of the 30 subendocardial MI’s, while LGE2 and LGE3 images identified the presence of infarction in 23 of the 30 subendocardial MI’s. MCODE identified 30 subendocardial infarctions.

Two patients had MI incorrectly identified by both MCODE and LGE. One of the two patients had Q waves in leads V1-V2 corresponding to a subendocardial anteroseptal region of LGE identified by both LGE and MCODE but no other confirmation of a clinically defined MI or significant coronary artery disease in a left anterior descending artery territory, and one patient had no corroborating evidence of MI, by clinical history, EKG, or other testing.

**Figure 3:**
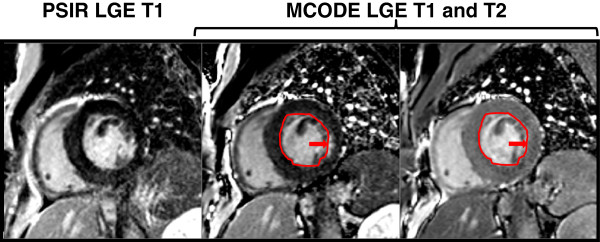
Example of an MCODE false positive MI: The above image is an example of one of MCODE’s false positive cases in a patient with an 80% circumflex stenosis but no clinical history of an MI or evidence of Q waves by EKG.

One MCODE false positive study did have angiographic evidence of significant coronary artery disease in the circumflex distribution and is displayed in Figure 3. On the LGE T1 image on the left, there is no overt MI. When one draws the subendocardial-blood pool border on the T2 image on the right and copies it to the T1 image, then one sees that what appears to be blood pool may actually be a focal subendocardial region of LGE. There was a corresponding regional wall motion abnormality.

#### Extent of infarction

**Figure 4:**
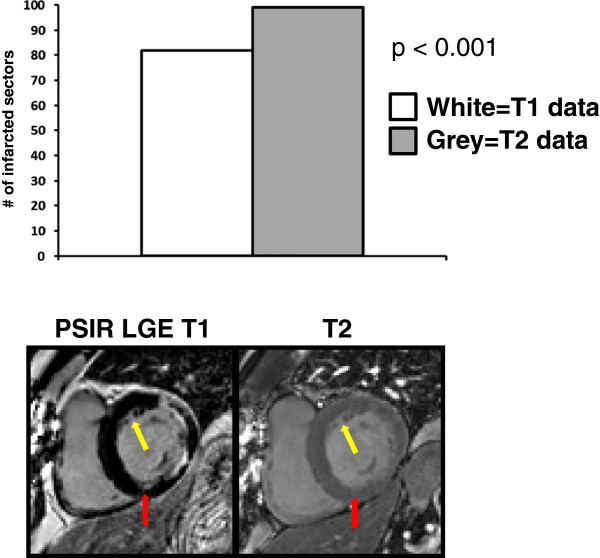
On a per sector basis, MCODE identified more infarcted sectors than LGE.

In sector-wise comparison of the two techniques, MCODE detected 99 sectors of infarction compared to 83 sectors detected by LGE2 (p < 0.001). Figure 4 illustrates an example in which an overt subendocardial MI of the anterolateral and inferolateral walls was identified by the PSIR LGE technique. However, MCODE aided in identifying an additional region of infarction within the inferior wall (red arrow). The MCODE T2 image also demonstrates that a similar small crevice adjacent to the anterior wall is blood pool (yellow arrow).

**Figure 5:**
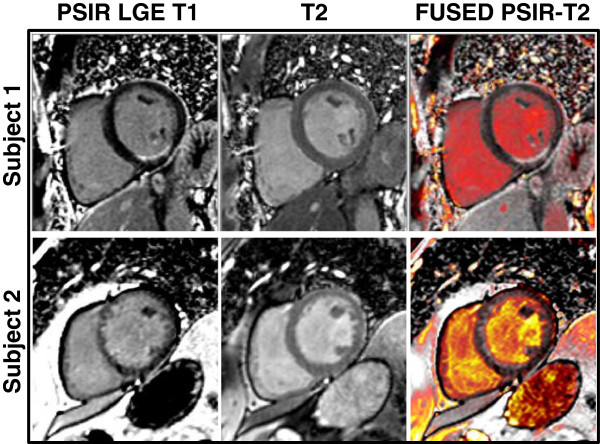
**Fusion of MCODE T1 and T2 data: DICOM images from MCODE sequences can be loaded into a free open source software, OsiriX Imaging Software.** From the MCODE sequence, the T2-weighted image is fused with the PSIR image with the Image Fusion function. No manual registration of the images is necessary since they were acquired during the same breath hold. When fused, the grey-scale T2-weighted image is then converted to the PET color look up table and overlays the grey-scale PSIR image. The image is then windowed and leveled to display areas with low T2 signal intensity (myocardium) to appear as dark red, and areas with high T2 signal intensity (blood pool) to appear as bright yellow.

Figure 5 displays a method of fusing the standard PSIR LGE T1 image (first column) with the T2 image (second column) to provide a composite image (third column) using commercially available software (OsiriX, Geneva, Switzerland). In the upper row, there is a clear cut subendocardial MI which is easily identifiable on the PSIR LGE image; MCODE does not add more information to this case. In the lower row, there is poor blood-pool-to-MI contrast. Use of the fused MCODE image provides clearer identification of the extent of the subendocardial MI along the anteroseptal and inferoseptal walls.

#### Comparison of signal intensities

**Figure 6:**
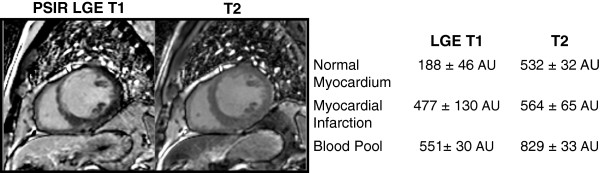
**Example comparison of LGE T1 and T2 signal intensity differences between normal myocardium, blood, and MI: In this example, LGE T1 signal intensities are similar between infarction and blood.** On the MCODE T2 image, both normal and infarcted myocardium have similar signal intensities but are different from the blood pool such that one can better differentiate where the endocardial border of the infarction is relative to the blood pool.

**Figure 7:**
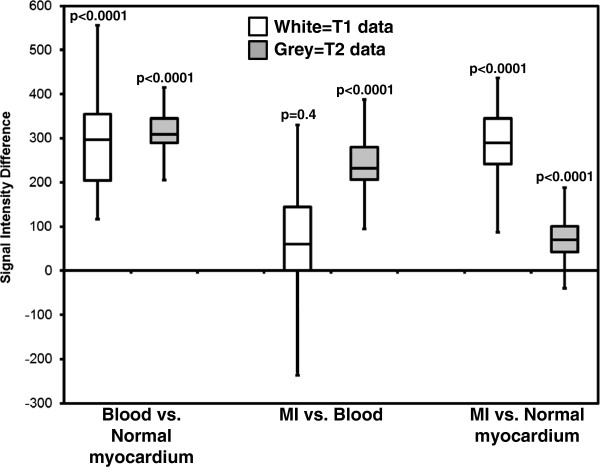
**Summary of signal intensity differences between blood, normal myocardium, and MI on LGE T1 and T2 images.** On T1 images, the MI to blood pool difference is not significant which can be diagnostically challenging. On the T2 images, there is a significant difference between the MI and blood pool signal intensities.

On MCODE T1 imaging, the signal intensity differences between MI and the blood pool were not statistically different (mean difference 16 arbitrary units (AU) ± 114, p = 0.4). On the T2 imaging, the signal intensity differences between MI and the blood pool were significant (mean difference 229 AU ± 70, p < 0.0001). In comparing acute MI T2 signal intensity differences of infarcted myocardium to blood pool against those of chronic MI’s, there was no significant difference (p = 0.09). Figure 6 demonstrates the different signal intensities between normal myocardium, MI, and the blood pool on both the LGE T1 image and the T2 images acquired with MCODE. Figure 7 displays the signal intensity comparison for all patients between MCODE, LGE, T1, and T2 images for blood vs. normal myocardium, MI vs. blood, and MI vs. normal myocardium comparisons. As hypothesized, there are significant differences between blood and normal myocardium on both T1 and T2 images (both p<0.0001). On LGE T1 images, there is very little difference between MI and blood, but more of a difference on the T2 images.

## Discussion

MCODE, a method that acquires both a LGE image and a T2-weighted image, improves detection of subendocardial MI and assessment of the extent of infarction compared with a conventional LGE image. While LGE and MCODE were equivalent in transmural MI, MCODE detected more subendocardial infarctions and found a greater extent of infarctions than conventional LGE. The main reason why MCODE improves the characterization of MI relates to the simultaneous acquisition of T1-weighted and T2-weighted images. Even in cases where there is poor contrast between the MI and the blood pool, the T2 image has excellent contrast between blood and myocardium. Thus, the simultaneous acquisition of LGE and T2-weighted images improves the ability to differentiate subendocardial contrast-enhanced infarcts because the T2-weighted image helps differentiate blood from myocardium.

Good blood pool to infarct/scar contrast depends upon multiple factors related to gadolinium kinetics: gadolinium dose, elimination rate of contrast, time between contrast agent administration and imaging. Additional factors that contribute to the ability to identify regions of LGE include its size, the image spatial resolution, and partial volume effects. If the issue were solely one of allowing enough time to pass to allow more clearance of gadolinium from the blood pool, then imaging at a later time frame after administration of contrast would solve the problem. Our study suggests that the issue is not dominated by timing between contrast injection and imaging. The bracketed LGE-MCODE-LGE experiment demonstrates that contrast timing washout does not explain why MCODE performs better than standard LGE. However, whether LGE works better at other time periods needs to be defined by future studies.

Particularly in the assessment of viability, answering the question of whether LGE is or is not present may not be enough. Clearly delineating the extent of an infarction may aid in targeting what vessels to revascularize. The patient displayed in Figure 4 had multi-vessel disease with right coronary artery dominance, documented by angiography, and by the initial T1 images, he had an overt anterolateral and inferolateral myocardial infarction, corresponding to the circumflex artery territory. The inferior myocardial infarction, which was detected only after using MCODE, corresponded to the right coronary artery. Although LGE may identify that an MI is present, MCODE not only adds confidence to the diagnosis, but it also may better identify the true extent of a MI.

While the present study is limited to evaluating patients with LGE in a MI pattern, there is prognostic significance affiliated with abnormal LGE patterns of either MI or atypical LGE. In a group of 857 patients with complete CMR cine and LGE exams, Cheong et al. demonstrated that abnormal LGE in both coronary artery disease and non-coronary artery disease patients was shown to predict all-cause mortality or cardiac transplantation [12]. Appropriate identification of any pattern of LGE may also assist in guiding management of patients with cardiomyopathy, as demonstrated by the study of Iles et al., in which 103 advanced cardiomyopathy patients underwent CMR imaging with gadolinium prior to implantation of an implantable cardioverter defibrillator. In one to two year follow-up, the study found that all defibrillator discharges occurred in both ischemic and nonischemic cardiomyopathy patients with abnormal LGE; whereas, none of the patients without LGE had any defibrillator discharges [13]. Kwong, et al. and others demonstrated that unrecognized MI as documented by LGE predicted worse future outcomes [3,14]. Thus, accurate recognition of LGE in a patient can make a clinical impact.

### Limitations

While the study design was carried out in a rigorous fashion, there were some limitations to the overall study.

One might argue that the SSFP cine image that is usually acquired in a complete CMR study could perform a function similar to the T2-weighted image in the MCODE image pair. This specific comparison was not performed in the current study, and therefore, it is difficult to know if the MCODE approach is superior to using the SSFP cine image without the direct comparison. However, multiple factors limit this comparison - the SSFP image and the T1 LGE image are acquired on separate breath-holds and at different time points, and there are often significant differences in slice positions or patient position, in temporal resolution and in trigger times, to mention a few.

While the study design did directly compare the standard LGE technique to the MCODE technique at similar times, the timing of the comparison was substantially later than the initial LGE acquisition. Therefore, the performance of MCODE, if applied at the same earlier time point as the initial LGE imaging, is unknown. In theory, at the earlier time point when the LGE blood-MI contrast is less, MCODE may have performed better.

A concern raised from this study’s findings might be that the standard LGE technique did not perform as well as expected compared to larger scale trials reporting the sensitivity and specificity of LGE in the diagnosis of MI [15]. However, it is important to note that only select slices in question underwent the bracketed LGE-MCODE-LGE design, and while on a specific slice comparison, the standard LGE technique may have missed an MI, in the context of reviewing an entire stack of LGE images with additional orthogonal views, the diagnostic performance of LGE likely improves. Furthermore, when comparing the current study’s performance to that of the large, multi-center double-blinded randomized trial assessing the performance of LGE using various doses of gadolinium, the current study’s sensitivity falls within the error bars of the 0.1 mmol/kg and the 0.2 mmol/kg doses’ performances.

Another recognized limitation is the fact that both standard LGE techniques and MCODE perform well in the diagnosis of MI. Therefore, large differences between the two techniques are difficult to detect in a study of this relatively smaller size.

## Conclusion

Our study demonstrates that in cases of subendocardial MI, the T2 data acquired by the MCODE sequence adds additional information to the final interpretation above that of the data obtained in a PSIR LGE T1 image alone. As more studies demonstrate the prognostic and clinical decision-making impact that LGE has on patients, CMR techniques need to also be able to more precisely identify LGE.

## Abbreviations

AU: Arbitrary units; CMR: Cardiovascular Magnetic Resonance; FLASH: Fast Low Angle Shot; Gd-DTPA: Gadolinium-diethylenetriamine pentaacetic acid; LGE: Late gadolinium enhancement; LGE1: Initial late gadolinium enhancement image; LGE2: Repeated late gadolinium enhancement image; LGE3: Third late gadolinium enhancement image; MCODE: MultiContrast Delayed Enhancement; MI: Myocardial infarction; NPV: Negative predictive value; PSIR: Phase sensitive inversion recovery; PPV: Positive predictive value; SD: Standard deviation.

## Competing interests

Dr. Arai is principal investigator on a US Government Cooperative Research and Development Agreement (CRADA) with Siemens.

## Authors’ contributions

AEA, WPB, and PK conceived of the study design and actively worked to coordinate details of the study, analysis of data, and drafting of the manuscript. CM, MYC, OJB, SV, and JRW were involved in the acquisition of data. SMS and SWL were involved in the acquisition of data and aided in statistical analysis. All authors were involved in the final editing of the manuscript and approve its content.
